# Comparing the Ability of Anthropometric Indicators in Identifying Metabolic Syndrome in HIV Patients

**DOI:** 10.1371/journal.pone.0149905

**Published:** 2016-02-26

**Authors:** Rebeca Antunes Beraldo, Gabriela Cristina Meliscki, Bruna Ramos Silva, Anderson Marliere Navarro, Valdes Roberto Bollela, André Schmidt, Maria Cristina Foss-Freitas

**Affiliations:** Department of Internal Medicine, Ribeirão Preto Medical School, University of São Paulo, Ribeirão Preto, São Paulo, Brazil; Temple University School of Medicine, UNITED STATES

## Abstract

**Background:**

Highly active antiretroviral therapy (HAART) can cause side effects in HIV patients, as the metabolic syndrome. Early identification of risk for development of cardiovascular diseases using available reliable and practical methods is fundamental. On this basis, the aim of this study was to compare the effectiveness of anthropometric indicators to identify metabolic syndrome in HIV patients on HAART.

**Methods:**

It is a cross-sectional study. A number of 280 stable HIV patients were studied. It measured weight, height, waist circumference (WC), hip circumference (HP), thigh circumference (TC) and calculated body mass index (BMI), body adiposity index (BAI), waist to hip ratio (WHR) and waist to thigh ratio (WTR). There was also a performance of biochemical tests of lipid profile and fasting glucose. Systemic blood pressure was measured. The criteria proposed by the National Cholesterol Education Program III (NCEP-ATP III) to metabolic syndrome classification was used. Individuals were divided in groups with or without metabolic alterations and their anthropometric indicators were compared. Receiver operating characteristic (ROC) curves were designed for each anthropometric indicator using the metabolic syndrome classification to identify sensitivity and specificity.

**Results:**

WC was a good tool to identify each metabolic disorder separately: total cholesterol (only females, p<0.05), triglycerides (only males, p<0.001), HDL cholesterol (p<0.05), LDL cholesterol (p<005) and fasting glycemic (p<005). WC also showed the best performance to identify metabolic syndrome in both genders (areas under the curve (AUCs): 0.79 and 0.76 for male and female, respectively), while BAI proved to be an inadequate indicator (AUCs: 0.63 and 0.67 for males and females), respectively, in this population.

**Conclusions:**

The central adiposity measure (WC) had the best performance to identify metabolic syndrome, and it is a convenient, cheap and reliable tool that can be used in clinical practice routinely to prevent cardiovascular complications in HIV patients.

## Introduction

The emergence and advances in highly active antiretroviral therapy (HAART) were the factors that increased HIV patients’ survival [1]. However, there are some specific toxicities of this therapy observed including morphological changes and lipid and glucose metabolism alterations [2]. Metabolic syndrome is a joint of interrelated risk factors of metabolic origin such as elevated blood pressure, glucose and lipid metabolism disturbances and obesity/central obesity, which are linked to the development of atherosclerotic cardiovascular diseases [3, 4].

Adipose tissue is still a major trigger of metabolic changes and development of chronic diseases and their redistribution is the central point of the metabolic syndrome. The determination of body composition is of great importance in clinical practice and the nutritional assessment of populations, mainly due to the association of body fat at risk for developing cardiovascular disease. Thus, this is important in the HIV group. [5,6]

Anthropometric indicators are considered simple, affordable and non-invasive methods that can be used in clinical practice to classify patients about the risk of diseases related to fat excess/redistribution [7]. The indicators are divided between those that enable the assessment of total fat and fat distribution [8].

Body Mass Index (BMI) is a total adiposity phenotype marker and it has been broadly studied in all age groups [9,10]. Among its limitations are the lack of ability to discriminate fat and lean mass and do not take into account the distribution of body fat [11]. Seeking transpose some limitations of BMI, in 2011 a new adiposity index was proposed, Body Adiposity Index (BAI) [12]. Several studies have evaluated its effectiveness for identifying metabolic diseases in different populations. However, investigations on the use to assess metabolic syndrome in HIV population are scarce [13].

Waist circumference (WC) is a central fat measurement while waist to hip ratio (WHR) evaluates the distribution of body fat. Both have been suggested as screening tools to identify individuals at risk of cardiovascular diseases [14].

Recently, our group proposed the waist to thigh ratio (WTR) for identifying lipodystrophy in HIV patients, however, its effectiveness to identify metabolic syndrome has not yet been compared to other anthropometric measures [15].

Several researchers have studied the use of fat indexes relating overweight to cardiovascular risk, but the use in HIV-infected population is still understudied [16–19].

As gain and changes in the distribution of body fat significantly increase morbidity and mortality in HIV patients on HAART, early identification of risk for development of cardiovascular diseases using available reliable and practical methods is fundamental. On this basis, the aim of this study was to compare the effectiveness of BMI, BAI, WHR, WC and WTR to identify metabolic syndrome in HIV patients on HAART.

## Materials and Methods

This is a cross-sectional study. It was conducted at the Outpatients Clinics of Ribeirão Preto Medical School (HC/FMRP) University Hospital.

Inclusion criteria were as follows: stable HIV patients (no signs or symptoms of opportunistic infections), use of HAART and stable weight (<10% change during the last year).

Exclusion criteria were as follows: presence of edema, thyroid disease, chronic renal insufficiency, pulmonary disease, hepatic alterations, signs or symptoms of opportunistic infections and presence of a pacemaker or a metal prosthesis.

All individuals who fulfilled the inclusion criteria, from January 2014 to October 2014 were invited to participate in the study.

The Research Ethics Committee of the Institution (HCRP protocol no. 17484 /2013) approved the study and all volunteer subjects gave written informed consent to participate.

Information was obtained about use of hypolipidemic and hypoglycemic drugs, currently used antiretroviral medications and biochemical examinations (viral load and T CD4 cells).

### Anthropometric Indicators

Trained staff performed all anthropometric measurements and at the same meeting. Before starting the evaluation, patients should remove all metal accessories and wear light clothing. They should empty the bladder and avoid the practice of rigorous physical activity in the previous 12 hours and consumption of alcohol in the 24 hours prior to the assessment of achievement.

Body weight, in kg, was measured with an electronic Filizola scale of the platform type with a maximum capacity of 300 kg and precision of 0.1 kg. Height was measured with a stadiometer with 0.1 cm precision. BMI was calculated as weight (kg) divided by height (m) squared.

The circumferences were performed using a metal measuring tape, Sanny, accurate to 0.1 cm and maximum length of 2m.

WC was performed midway between the inferior margin of the last rib and the crest of the ilium in a horizontal plane. Hip circumference (HC) was performed in the region of largest circumference between the waist and the thigh. Thigh circumference (TC) was performed in the end of the right gluteus [20].

BAI was calculated from the HC (cm) divided by height (m) high power 1.5 and subtracted 18 [(HC) / (height ^1.5^)]– 18. WHR was calculated from WC (cm) divided by HC (cm). WTR was calculated from WC (cm) divided by TC (cm). [12,15].

### Blood Analysis

Lipids (triglyceride (TGs), total cholesterol (TC), and high-density lipoprotein (HDL)) concentrations were measured by enzymatic colorimetric method on COBAS INTEGRA 400 instrument (Roche Diagnostics^,^ Indianapolis, IN, USA) in the blood. Low-density lipoprotein LDL was calculated with the Friedewald formula [LDL = TC—(TGs/5 + HDL)]. Plasma glucose was analyzed with a Yellow Springs Instruments 2300 STAT Glucose Analyzer (Yellow Springs Instruments Inc., Yellow Springs, OH, USA).

### Metabolic Syndrome

The criteria suggested by The National Cholesterol Education Program III for risk of metabolic syndrome were used (total cholesterol ≥220mg/dl, triglycerides ≥150mg/dl, HDL cholesterol ≤40mg/dl, LDL cholesterol ≥130mg/dl or treatment for dyslipidemia; fasting glycemia ≥100 mg/dl or treatment; systolic blood pressure ≥130mmHg and diastolic blood pressure ≥85mmHg or treatment for hypertension; WC ≥88cm for females and ≥102cm for males). Metabolic syndrome was considered when the patient had three or more items [21].

### Statistical Analysis

The continuous variables are reported as means, s.d., and the categorical variables as frequencies and percentages.

Individuals were divided into different groups: Cho-, Cho+, Tri-, Tri+, HDL-, HDL+, LDL-, LDL+, gly-, gly+, MS-, MS+ (according to The National Cholesterol Education Program III for risk of metabolic syndrome).

Comparisons between patients Cho- and Cho+, between Tri- and Tri+, between HDL- and HDL+, between LDL- and LDL+, between gly- and gly+ and between MS- and MS+ were made by Student’s t-test (continuous variables) and a χ2-test (categorical variables).

Receiver operating characteristic (ROC) curves were designed for each anthropometric indicator using the metabolic syndrome classification to identify sensitivity and specificity. The determination of the cutoff points was based on the values that maximized simultaneously both sensitivity and specificity.

Analyses were carried out using the BioEstat 5.3. A 5% significance level was considered in all analysis.

## Results

The final sample consisted of 280 stable HIV-seropositive patients under HAART (57.14% male and 42.85 female). Mean age was 44.09 ± 10.13 years, mean time of positive serology was 10.01 ± 6.86 years, and mean time of HAART use was 7.99 ± 6.12 years.

Regarding HAART regimen type, 51.1% of the patients were taking a combination of two nucleoside analog reverse transcriptase inhibitors (NRTI) with a non-nucleoside analog reverse transcriptase inhibitor (NNRTI); 41.4% were taking a combination of two NRTIs with a protease inhibitor (PI); 3.2% were taking a combination of a NRTI, a NNRTI and a PI; 3.6% were taking a combination including an integrase inhibitor (II); and 0.7% were taking a combination including a fusion inhibitor (FI).

Most individuals had dyslipidemia, as 27.1% had hypercholesterolemia, 42.5% had hypertriglyceridemia, 57.5% had reduced HDL, and 31.4% had increased LDL. However, only a small proportion of individuals (21.0%) was taking oral lipid-lowering medications such as statins and fibrates. Additionally, 28.1% of the patients had a diagnosis of systemic arterial hypertension (SAH), 22.5% had altered blood pressure during the evaluation and 20.7% was taking anti-hypertension drugs. Furthermore, 31.4% had altered fasting glycemia and 8.9% had the diagnosis of diabetes mellitus (DM), but only 7.5% was taking oral antihyperglycemic agents and only 2.8% was taking insulin.

The mean weight of the population studied was 70.8 ± 16.4kg, mean height was 165.5 ± 8.8cm.

The individuals had a high BMI (25.8 ± 5.6 kg / m²). WC and WHR were on average increased in females (94.1 ± 15.0 cm and 0.93 ± 0.08), but appropriate in males (92.2 ± 13.4 cm and 0.95 ± 0.08). According to BAI, men and women were on average within the recommended limits of 25 and 35 for both males and females, respectively (24.8 ± 4.4 and 32.6 ± 6.6, respectively). However, Table 1 indicates that the cutoff points to identify metabolic syndrome of the population evaluated in this study may differ from those recommended for the seronegative population.

**Table 1:** Cutoff point to identify metabolic syndrome, sensitivity, specificity, positive and negative predictive value for each anthropometric indicator separated by gender (data obtained from the ROC curves).

	Cutoff point	Sensitivity	Specificity	Positive predictive value	Negative predictive value
Body mass index (male)	25.38 kg/m^2^	67.9%	68.1%	80.2%	73.3%
Body mass index (female)	25.27 kg/m^2^	70.8%	72.2%	73.8%	67.2%
Body adiposity index (male)	24.49	61.5%	64.2%	62.3%	63.3%
Body adiposity index (female)	31.16	66.6%	68.2%	70.3%	62.5%
Waist to hip ratio (male)	0.97	71.8%	79.0%	76.7%	74.3%
Waist to hip ratio (female)	0.93	65.2%	72.2%	74.1%	62.9%
Waist circumference (male)	95 cm	70.5%	76.6%	83.3%	78.7%
Waist circumference (female)	90 cm	84.8%	64.8%	74.6%	77.7%
Waist to thigh ratio (male)	1.74	76.9%	68.3%	69.8%	75.6%
Waist to thigh ratio (female)	1.59	75.8%	63.0%	71.4%	68.0%

It was observed the metabolic syndrome in most (51.42%) of the patients (78 males and 66 females).

Central fat and fat distribution indicators (WC, WHR and WTR) were more efficient than BMI and BAI to identify each altered metabolic parameter (Table 2).

**Table 2:** Mean values of each anthropometric indicator according to metabolic parameters separated by gender. Data are presented as mean ± Standard deviation (number of individuals).

	BMI		BAI		WHR		WC		WTR	
	M	F	M	F	M	F	M	F	M	F
Cholesterol										
Cho -	24.9 ± 4.9 (118)	26.0 ± 6.1 (92)	24.9 ± 4.6	32.0 ± 6.2	0.94 ± 0.08	0.92 ± 0.08	90.9 ± 13.8	92.2 ± 15.0	1.73 ± 0.21	1.65 ± 0.20
Cho+	25.8 ± 4.2 (42)	28.9 ± 7.5* (28)	24.7 ± 3.7	34.4 ± 7.8	0.99 ± 0.07*	0.97 ± 0.09*	95.6 ± 11.5	100.1 ± 14*	1.83 ± 0.18*	1.76 ± 0.19*
Triglycerides										
Tri -	24.2 ± 5.4 (76)	26.1 ± 5.9 (75)	25.0 ± 5.4	32.3 ±6.1	0.92 ± 0.08	0.91 ± 0.08	88.6 ± 15.1	92.3 ±14.5	1.70 ± 0.22	1.64 ± 0.21
Tri+	25.8 ± 3.8* (84	27.6 ± 7.5 (45)	24.7 ± 3.2	33.0 ± 7.6	0.98 ±0.07**	0.96 ± 0.08*	95.4 ± 10.7**	97.0 ± 15.8	1.81 ± 0.17**	1.73 ± 0.18*
HDL Cholesterol										
HDL-	25.6 ± 4.1(81)	27.2 ± 6.6 (94)	24.9 ± 3.8	32.9 ± 6.8	0.97 ± 0.08	0.94±0.08	94.0 ± 12.8	95.5 ± 14.7	1.78 ± 0.19	1.69 ± 0.20
HDL+	24.5 ± 5.3(79)	24.8 ± 6.3 (26)	24.9 ± 5.0	31.4 ± 5.8	0.94 ± 0.08*	0.89±0.09*	90.2 ± 13.9*	89.1 ± 15.8*	1.74 ± 0.22	1.64 ± 0.22
LDL Cholesterol										
LDL-	24.5 ± 4.4 (98)	25.6 ± 6.1(77)	24.7 ± 4.1	31.5 ± 6.1	0.94 ± 0.08	0.92 ± 0.09	89.9 ± 13.1	91.7 ± 15.1	1.73 ± 0.19	1.65 ± 0.22
LDL+	26.0 ± 5.1 (62)	28.6 ± 6.9 (43)	25.1 ± 4.9	34.5 ± 7.2*	0.98 ± 0.08	0.95 ± 0.08	95.7 ± 13.4*	98.4 ± 14.2*	1.81 ± 0.22*	1.71 ± 0.18
Fasting glycemia										
Gly-	24.5 ± 4.2 (101)	5.8 ± 6.2 (84)	24.8 ± 3.7	32.1 ± 6.3	0.94 ± 0.08	0.92 ± 0.09	89.9 ± 12.3	91.5 ± 14.7	1.71 ± 0.18	1.64 ± 0.20
Gly+	26.1 ± 5.4 (59)	28.8 ± 6.9* (36)	24.9 ± 5.5	33.8 ± 7.3	0.98 ± 0.08*	0.96 ± 0.07*	96.0 ± 14.6*	100.3 ± 14.5*	1.83 ± 0.21**	1.75 ± 0.20*
Metabolic syndrome										
MS-	22.8 ± 3.13 (82)	23.8 ± 5.8 (52)	23.7 ± 3.26	30.3 ± 5.9	0.91 ± 0.07	0.89 ± 0.08	84.8 ± 9.2	86.5 ± 14.4	1.67 ± 0.17	1.61 ± 0.22
MS+	27.4 ± 5.0**(78)	29.0 ± 6.2**(66)	26.0 ±5.16*	34.5 ±5.9**	1.00 ± 0.07**	0.96 ± 0.08**	99.8 ± 12.9**	100.4 ±12.6**	1.86 ± 0.19**	1.73 ± 0.17**

BMI: body mass index; BAI: body adiposity index; WHR: waist to hip ratio;WC: waist to hip circumference; WTR: waist to thigh ratio; M: male; F: female; Cho- and Cho+: patients with total cholesterol lower and higher than 220 mg/dl, respectively; Tri- and Tri+: patients with triglycerides lower and higher than 150 mg/dl, respectively; HDL- and HDL+: patients with HDL cholesterol lower and higher than 40 mg/dl (male) and 50 mg/dl(female), respectively; LDL- and LDL+: patients with LDL cholesterol lower and higher than 130 mg/dl, respectively, gly- and gly+: patients with fasting glycemia lower and higher than 100 mg/dl, respectively; MS- and MS+: patients with or without metabolic syndrome according to The National Cholesterol Education Program III criteria, respectively.

* p<0.05;

**p<0.001 comparing groups—with + for each gender.

**Fig 1:**
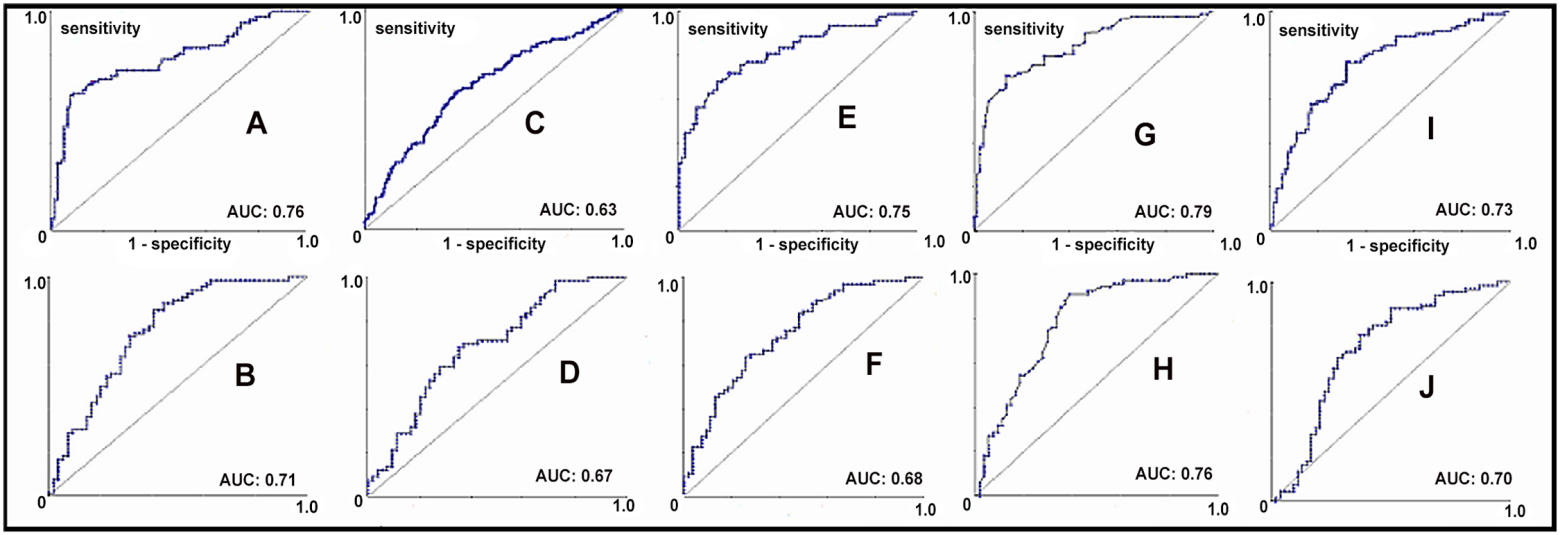
Receiver operating characteristic curves for the performance evaluations of anthropometric indicators in identifying metabolic syndrome. The performance of the test evaluated by the area under the curve (AUC) in body mass index (BMI) for males (A), BMI for females (B), body adiposity index (BAI) for males (C), BAI for females (D), waist to hip ratio (WHR) for males (E), WHR for females (F), waist circumference (WC) for males (G), WC for females (H), waist to thigh ratio (WTR) for males (I) and WTR for females (J).

WC showed the best performance to identify metabolic syndrome in both genders (areas under the curve (AUCs): 0.79 and 0.76 for male and female, respectively), while BAI proved to be an inadequate indicator (AUCs: 0.63 and 0.67 for males and females, respectively) for identifying metabolic disorders in this population (Fig 1).

## Discussion

In this study, we evaluated the effectiveness of simple tools to assist in the early evaluation of the metabolic syndrome. This is an original study and to our knowledge, no previous report has compared anthropometric measures to indicate metabolic syndrome in a large population of HIV patients on HAART. As our main result, we found that the anthropometric indicators of central fat / fat distribution (WC, WHR, WTR) were more suitable to identify metabolic changes in this population.

Previous studies only correlated anthropometric ratios with the gold standard dual-energy X-ray absorptiometry (DXA) [22,23]. However, none had assessed what ratio has the best performance to indicate metabolic disorders in a large population with both sexes.

We found that most of the patients presented metabolic syndrome, indicating a high risk of developing cardiovascular disease. These data are worrisome because of the high incidence of acute myocardial infarction and stroke in this population.

Our study confirms the hypothesis that intra-abdominal fat tissue accumulation is associated with metabolic and neuroendocrine disorders [24], as the WC measurement showed the best effectiveness for evaluation of metabolic syndrome in both genders. Several studies have found that WC has been the best of the anthropometric indicators, with excellent correlation to abdominal imaging and high association with cardiovascular risk factors, especially diabetes [8,25,26]. WC evaluates the fat deposition in the abdominal region, and abdominal visceral obesity is a more severe cardiovascular risk factor than general obesity [27–29] that is usually assessed by BMI.

Abdominal adipose tissue synthesizes and secretes several mediators and cytokines, including tumor necrosis factor α (TNF α), intracellular carriers of glucose (GLUTs), gamma receptors activated by peroxisome proliferator (PPARγ) and resistin, that participate in mechanisms that lead to dyslipidemia, insulin resistance, hypertension and atherosclerosis. It is also known that the adipocyte, according to its location, has different metabolic characteristics, and the intra-abdominal adiposity is the one with the greatest impact on the deterioration of insulin sensitivity [30,31].

On the other hand, the gluteal-femoral subcutaneous adipose tissue appears to exert a protective function concerning insulin resistance. Thus, in the case of redistribution of body fat, the two factors (lipoatrophy and lipo hypertrophy) end up contributing to a worsening of the metabolic profile. Thinking about it, Beraldo et al.[15] proposed WTR that evaluate the relation between central fat (waist)—peripheral fat (thigh). The index showed a good performance to identify increased cholesterol, triglycerides, LDL (only in males), blood glucose and presence of metabolic syndrome, though not surpassed WC. The results for WHR and WTR were very similar because both evaluate the same fat distribution (central-peripheral). It was expected a better performance of WTR due to these patients have a higher loss of fat in the thighs than in the hip, but this was not found.

BMI was a bad tool to identify each metabolic change separately. However, their performance has been good at identifying the metabolic syndrome as a whole (Fig 1A and 1B), but not exceeding WC. Despite being an anthropometric indicator of total fat, a study of the HIV population found that BMI has a high correlation with the trunk fat (measured by DXA), and this correlation was even greater than WHR correlation. The specificity of the body composition of the investigated group, as the excessive accumulation of fat in the trunk region (lipo hypertrophy) or the little amount of fat in the limb areas (lipoatrophy), should handle the high rates of correlation between BMI and trunk fat mass [13]. However, in spite of this particularity in HIV patients, we found a bad performance to identify changes in total cholesterol, triglycerides (females), LDL, HDL and fasting glucose (males).

In our study, BAI did not appear as a good tool to identify metabolic changes. BAI has been proposed with the intention of crossing the limitations of BMI; however since it was proposed, and several studies in various populations with different ethnicities have shown that it is not a very accurate index.

BAI has a tendency to overestimate adiposity at lower percentage body fat and underestimate adiposity at higher percentage body fat [32]. Additionally, although BAI appeared to be better than BMI to correlate with body fat (measured by DXA) when both genders were considered together it does not occur when males and females are separated. This may explain the findings of our and others studies that have found that BAI was not better than BMI or WHR in identifying people with metabolic disorders [33–36].

WTR was the only one that the cutoff was similar than that established in a previous study for males (1.74) due to it was developed in HIV population [15]. Cutoff points had not been established for females, and our results suggest that they can be smaller than the male cutoff.

All other anthropometric indicators have different values from the proposed for the seronegative population. The cutoff points proposed by the World Health Organization for BMI (≥24,9kg / m^2^), WC (≥94cm for males and ≥80cm for females) and WHR (≥1 for males and ≥0,85 for females) were drawn up in the Caucasian population [37]. The cutoff points for BAI have been proposed in the North American population [12]. Since then, these cut-points have been used as a standard in different populations and different ethnic groups, with the assumption these different groups is at similar cardiovascular risk. However, questioning their applicability in various ethnic groups, several recent studies have found different points to their specific populations [38–45]. Our study did not aim to propose new cutoff points since the sample size appears not suitable for this, but our results indicate a possible difference and suggests that further works should be carried out to confirm this hypothesis.

We have chosen to separate patients according to gender because of the difference in body fat amount and distribution. Women have a higher percentage of body fat than men do. In addition, women store more fat in the gluteal-femoral region, whereas men store more fat in the visceral (abdominal) depot [46]. We observed that males performance of almost all anthropometric indicators (except BAI) to identify metabolic syndrome was slightly higher than in females.

Finally, our results are of great importance as the central adiposity measure (WC) showed the best performance to identify metabolic syndrome in HIV patients. It is a convenient cheap and reliable tool that can routinely be used in clinical practice. Another important finding is that our results indicate a possible difference in the cutoff points for anthropometric indicators in HIV patients.
